# Perceptions and attitudes around perinatal mental health in Bangladesh, India and Pakistan: a systematic review of qualitative data

**DOI:** 10.1186/s12884-022-04642-x

**Published:** 2022-04-06

**Authors:** Nafisa Insan, Anthony Weke, Judith Rankin, Simon Forrest

**Affiliations:** 1 Population Health Sciences Institute, Newcastle University, Newcastle Upon Tyne, UK; 2 Department of Sociology, Durham University, Durham, UK

**Keywords:** Perinatal mental health, South Asia, Perceptions, Attitudes

## Abstract

**Background:**

Perinatal mental health (PMH) is a worldwide public health issue crossing cultural boundaries. However, the prevalence of PMH conditions vary considerably. These disparities stem in part from poor understanding and stigma surrounding PMH which hinder pregnant women from seeking mental health care and may exacerbate their conditions. Bangladesh, India and Pakistan are South Asian countries with a higher burden of PMH conditions than in the Global North-West and very different social and cultural norms around gender and mental health. The aim of this systematic review (PROSPERO Ref: CRD42020167903) was to identify, synthesise and appraise the available literature on perceptions and attitudes of perinatal (pregnant and postpartum) women, their families and healthcare providers surrounding PMH in Bangladesh, India and Pakistan.

**Methods:**

Five electronic databases, MEDLINE, Embase, PsycINFO, Scopus and Web of science, and grey literature were searched using predefined search terms. Qualitative or quantitative articles with a qualitative component reporting perceptions and attitudes surrounding PMH in Bangladesh, India and Pakistan were eligible for inclusion, if published in English between January 2000 and January 2021. The Critical Appraisal Skills Programme Qualitative Research Checklist and Newcastle–Ottawa Scale for cross-sectional studies were used to assess study quality. Findings were synthesised using thematic synthesis, as described by Thomas and Harden 2008.

**Results:**

Eight studies were included. Five overarching themes comprising 17 sub-categories were identified. These descriptive themes were: perceived causes of PMH, perceived symptoms of PMH, perceptions of motherhood, accessing PMH care and emotional sharing and coping strategies. Sociocultural expectations underpin many of the themes identified in this review including the importance of familial and societal causes of PMH, emphasis on physical symptoms, sacredness of motherhood, lack of awareness, stigma, shame, limited resources allocated for mental health and lack of emotional sharing.

**Conclusions:**

There is a complex range of perceptions and attitudes around PMH which influence women’s experiences and access to PMH care. These findings will inform policy and practice through targeted interventions to tackle stigmatising attitudes and increasing education and training for healthcare providers.

**Supplementary Information:**

The online version contains supplementary material available at 10.1186/s12884-022-04642-x.

## Background

Perinatal mental health (PMH) conditions, particularly depression and anxiety being the most common PMH conditions, have been long considered a Western biomedical construct [1]. It was thought that the rise in modern obstetric practices in Western countries, which isolated women from guidance and social support throughout their pregnancy and after childbirth, was related to maternal depression [2]. This medicalisation of pregnancy often shifted the care of women away from sociocultural practices such as mandated rest and social recognition of new social status through rituals and gifts into more professional healthcare settings. Hence, it was assumed that women from non-Western countries were protected from depression by these culturally specific rituals and factors [2]. However, there is little evidence to support this. Not only do PMH conditions such as depression seem to occur universally, but recent research has investigated the cross-cultural validity of maternal depression and has found there to be a convergent validity for the construct of maternal depression in terms of the emotional and somatic distress in the setting of social adversity [3]. Therefore, PMH is a worldwide public health issue crossing cultural boundaries. However, conceptualisation of perinatal depression and anxiety as a psychiatric, or even a medical disorder is less applicable in South Asian cultures which have differing perceptions of PMH making it a challenging, yet important, construct to tackle in these cultural settings [3, 4].

Bangladesh, India and Pakistan are South Asian countries which have very different social and cultural norms to Western countries. Previous literature has found the prevalence of perinatal depression in these South Asian countries to range from 10% [5] to as high as 39% [6], compared to 7–15% in High Income Countries (HICs) [7]. These disparities stem from the poor understanding and high levels of stigma around mental health conditions in South Asia [8], which prevent pregnant women from seeking and accessing mental health care and reporting mental health issues due to shame and fear of judgment. It also impacts the way families and healthcare providers of pregnant women manage and view depression during pregnancy, consequently, affecting the care women receive. It is, therefore, imperative we investigate the perceptions and attitudes that these communities in Bangladesh, India and Pakistan have towards PMH to aid our understanding of why these countries have high rates of perinatal depression, what needs to be done to break the stigma and to make mental health care more available and accessible for pregnant women.

Perceptions and attitudes, which are heavily contingent and linked to socio-cultural context, influence how individual’s understand mental illness and the way they respond to situations, activities and relationships [9]. South Asian cultures, like Bangladeshi, Indian and Pakistani, are very collectivist, in that social controls such as family, culture, religion and community, often dominate decision-making and strongly shape the attitudes that individuals hold [10]. Hence, the way such collectivist cultures perceive social roles and conceptualise identities will influence individuals’ perceptions and attitudes towards certain phenomena such as PMH. A systematic review by Watson et al. [11] investigating ethnic minority women’s, largely South Asian and Black African, experience of PMH conditions and services in the UK found that lack of awareness, ongoing stigma and cultural expectations all impacted women’s receipt of adequate PMH support in the UK [11]. However, there is limited research investigating these experiences in Bangladesh, India and Pakistan where the social and structural settings are vastly different.

To our knowledge, there are no existing systematic reviews which have investigated perceptions and attitudes of perinatal (pregnant and postpartum) women, their families and health care providers towards PMH in Bangladesh, India and Pakistan. These groups are key actors in perinatal women receiving mental health care. Therefore, the aim of this systematic review is to identify, synthesise and appraise the available literature on perceptions and attitudes of perinatal women, their families and healthcare providers surrounding PMH in Bangladesh, India and Pakistan. This will inform the development of PMH services in these countries and interventions and programmes to improve mental health of perinatal women.

## Methods

This systematic review followed the Enhancing Transparency in Reporting the synthesis of qualitive research (ENTREQ) guidelines (See Additional File 1).

### Search strategy

Inclusion criteria were qualitative and quantitative studies with a qualitive component reporting perceptions and attitudes related to women in the perinatal period, their families and healthcare providers surrounding PMH (only including perinatal depression and anxiety as the most common PMH conditions). Studies must be conducted on populations in Bangladesh, India or Pakistan and be published in English between 1^st^ January 2000 and 31^st^ January 2021. This date restriction was imposed because scoping searches on Medline showed no appropriate studies before 2000, suggesting that investigation of maternal depression and anxiety in South Asia is a relatively recent area of research enquiry. Over time, clinical approaches and thinking towards maternal depression and depression as a whole, particularly in Low and Middle Income countries (LMICs), have developed and changed [3]. Therefore, this date restriction ensured that the literature in this review would still be clinically and socially relevant. Studies were excluded if they had no qualitative element that could be extracted, not conducted in Bangladesh, India or Pakistan and published outside of the time frame investigated.

A systematic literature search was conducted via the electronic databases MEDLINE, Embase, PsycINFO, Scopus and Web of science using keywords and MeSH headings developed with an information specialist (See Additional File 2 for full OVID search strategy). This review relating to perceptions and attitudes forms part of a wider search strategy which also included a second review investigating the social determinants of antenatal depression in South Asia [12] (see PROSPERO Ref: CRD42020167903). Search results were exported into the reference manager Zotero and duplicates were removed. Titles and abstracts were independently screened by two reviewers (NI and AW) and compared. Those that met the criteria were obtained in full and assessed by the research team to determine inclusion. Grey literature was searched using the Open Grey database and also via charities and non-governmental organisations such as Building Resources Across Communities (BRAC), UNICEF and National Health Mission India. Supplementary searches included hand searching study reference lists included from databases and citation searches using Google Scholar. Database searches were completed on 31^st^ January 2020 and updated on 4^th^ January 2021.

### Quality appraisal

Quality appraisal was carried out by two reviewers (NI and AW) and agreement was reached through discussion. Study quality was assessed using the 2018 Critical Appraisal Skills Programme (CASP) checklist for qualitative studies (see Additional File 3) [13]. A scoring of 1 mark for each question that was answered with “Yes” was allocated to provide an indicator of quality and enabled comparison between reviewers. Cut-off scores of $\ge$ 9 indicated ‘strong’ quality, 6–8 indicated ‘adequate’ quality and $\le$ 5 indicated ‘weak’ quality for the CASP tool.

For the primarily quantitative studies included in this review, there are no CASP checklists for cross-sectional studies, therefore, the Newcastle–Ottawa Scale (NOS) adapted for cross-sectional studies which assesses information bias, selection bias and confounding bias was used to assess quality (see Additional File 4) [14]. The maximum quality score a paper could receive was 10; 0–3 reflected ‘low’ quality papers, 4–7 was ‘medium’ and 8–10 indicated ‘high’ quality.

### Data extraction and synthesis

Data including bibliographic information, country, type of study, sampling size and method, participant characteristics, data collection method, data analysis method, quality assessment and findings were recorded on a standardised data extraction form. The integrated mixed methods synthesis approach developed by Sandelowski et al. [15] was adopted. This involves transforming the data which are then integrated and synthesised together. In this review, the data from the cross-sectional studies were transformed into qualitative data, as the studies used surveys or semi-structured interview questionnaires that consisted of phrases/sentences that summarised certain attitudes and perceptions. The transformation was done by extracting the statements that were mostly agreed with by the participants and that the authors highlighted in their findings and discussion and using those as qualitative statements to form themes. This method is appropriate when both quantitative and qualitative studies address the same research question.

Integration and synthesis of qualitative findings were carried out using thematic synthesis adapted from Thomas and Harden [16] by the lead reviewer (NI) in two iterative stages: 1) coding of extracted data: data from included studies were examined for meaning and content and codes were applied to capture these. As each new study was examined, similar codes were highlighted, and new codes were added to the bank; 2) Grouping of codes into themes: list of initial codes were analysed for meaning and organised into categories of similar or associated meanings. Each category was examined, and descriptive themes were generated and relationships between them were revealed. Themes were then interrogated by other reviewers (SF and JR).

## Results

### Included studies

**Fig. 1:**
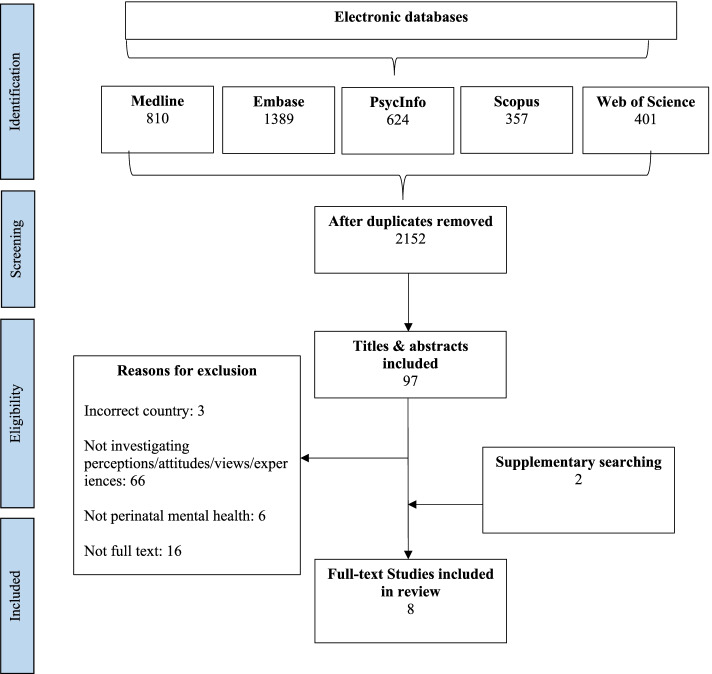
PRISMA Flow diagram

Eight studies published between 2000 and 2021 met the inclusion criteria (Table 1). See Fig. 1 for the PRISMA flow diagram. Six studies were conducted in India [3, 17–21], two in Bangladesh [9, 22] and one in Pakistan [19]. Four studies were qualitative [3, 9, 19, 22] and three were cross-sectional studies [17, 18, 20, 21]. Sample sizes ranged from 21 to 332 using mostly purposive or convenience sampling. The included studies investigated perceptions and attitudes from four population groups: pregnant women [17–19, 21], postpartum women [3, 9, 17, 19, 21, 22], family members of pregnant women [3, 20] and healthcare providers [9, 21]. The CASP quality appraisal of qualitative studies found that all four qualitative studies [3, 9, 19, 22] were of ‘adequate’ quality. The NOS quality appraisal of cross-sectional studies found that three studies [17, 20, 21] were of ‘medium’ quality and one study [18] was of ‘low’ quality.

**Table 1:** Summary of Included studies

Author, publication year	Country	Aims	Type of study, Sampling size & method	Participant characteristics	Data collection method	Data analysis method	Main themes and sub-themes identified	Quality
Edhborg et al., 2015	Bangladesh	“To explore and describe the experiences and concerns during the first 3–9 months following childbirth of those mothers who showed depressive symptoms 2–3 months postpartum, in a rural area in Bangladesh”	Qualitative 21 Purposive sampling	Mothers with depressive symptoms 2–3 months postpartum	Narrative interviews	Inductive content analysis	Perceived causes of PMH (Gender of baby, IPV, Relationship with husband/in-laws, economic difficulties, spiritual) Perceived symptoms of PMH (emotional, physical) Baby’s health is most important Awareness of PMH is an enabler of accessing PMH care	Adequate^b^
Goyal et al., 2020	India	“To study psychiatric morbidity, its prevalence and cultural factors influencing illness understanding, help-seeking behaviour and barriers to care in perinatal women.”	Mixed methods (cross-sectional with a qualitative component) 27 Purposive sampling	Perinatal women with depression or anxiety screened using EPDS or PASS	Cultural Formulation Interview	Thematic interpretations	Perceived causes of PMH (spiritual) Perceived symptoms of PMH (emotional, physical) Barriers to accessing PMH care (stigma, limited resources) Friendly and confidential counselling are enablers to accessing PMH care Religious and personal coping strategies	Medium^a^
Manjrekar et al., 2018	India	“To find out the awareness and perception of mental health problems in pregnant women residing in rural areas of India”	Cross-sectional 300 Convenience sampling	Antenatal women	Semi structured questionnaire interview	Descriptive statistics	Perceived causes of PMH (spiritual) Barriers to accessing PMH care (stigma, limited resources) Friendly and confidential counselling are enablers to accessing PMH care	Low^a^
McCauley et al., 2020	India & Pakistan	“To explore what women attending for routine antenatal care (ANC) or postnatal care (PNC) at healthcare facilities in India and Pakistan consider health and ill health to be in general, and, what they consider health and ill health to be, during and after pregnancy”	Qualitative 130 Purposive sampling	Antenatal and postnatal women	Focus group discussions	Thematic framework analysis	Perceived causes of PMH (Gender of baby, IPV, Relationship with husband/in-laws) Perceived symptoms of PMH (emotional) Baby’s health is most important Barriers to accessing PMH care (stigma, limited resources) Enablers to accessing PMH care (awareness, friendly and confidential counselling)	Adequate^b^
Poreddi et al., 2020	India	“To explore the knowledge and attitudes of family members towards postpartum depression.”	Cross-sectional 202 Random sampling	Family members of postpartum women	Face to face interview using semi-structured questionnaire	Descriptive statistics	Perceived causes of PMH (Lack of practical support, Gender of baby, IPV, economic difficulties, spiritual) Perceptions of motherhood (baby’s health and motherhood are sacred) Barriers to accessing PMH care (stigma) Enablers to accessing PMH care (friendly and confidential counselling)	Medium^a^
Ransing et al., 2020	India	“To assess the knowledge gap, perceptions, and misconceptions about perinatal depression at three different levels i.e. high-level service providers (e.g. specialists, general practitioners), mid-level health care providers (nurses, midwives) and service utilizers (perinatal women).”	Cross-sectional 332 Convenience sampling	270 Perinatal women 42 Nursing providers 20 Medical practitioners	Questionnaire and online survey forms	Descriptive statistics	Perceived causes of PMH (Lack of practical support, Gender of baby, relationship with husband/in-laws) Perceived symptoms of PMH (emotional) Motherhood is sacred Barriers to accessing PMH care (stigma) Enablers to accessing PMH care (awareness, friendly and confidential counselling)	Medium^a^
Rodrigues et al., 2003	India	“To describe attitudes and perceptions of mothers and husbands towards childbirth in Goa in order to explore the processes through which the relationship between social adversity and PND is mediated.”	Qualitative 39 Purposive sampling	Postpartum mothers (19 with postpartum depression) and their husbands	In-depth interviews	Thematic analysis	Perceived causes of PMH (Lack of practical support, Gender of baby, IPV, relationship with husband/in-laws, economic difficulties) Perceived symptoms of PMH (physical, emotional) Baby’s health is most important Barriers to accessing PMH care (stigma, limited resources)	Adequate^b^
Williams et al., 2018	Bangladesh	“To understand the cultural attitudes, from both new mothers and maternal health professionals, towards mental health post-childbirth.”	Qualitative 70 Purposive sampling	36 postpartum mothers 34 medical personnel	In-depth interviews	Thematic analysis	Perceived causes of PMH (Lack of practical support, IPV, economic difficulties) Perceived symptoms of PMH (physical) Perceptions of motherhood (baby’s health and motherhood are sacred) Barriers to accessing PMH care (stigma, limited resources)	Adequate^b^

*EPDS* Edinburgh Postnatal Depression Scale

*PASS* Perinatal Anxiety Screening Scale

^a^Quality assessed using Newcastle–Ottawa Scale for cross-sectional studies (see Additional File 4)

^b^Quality assessed using the CASP tool for qualitative studies (see Additional File 3)

### Findings

Five themes were synthesised concerning perceptions and attitudes surrounding PMH in Bangladesh, India and Pakistan: (1) Perceived causes of PMH, (2) Perceived symptoms of PMH, (3) Perceptions of motherhood, (4) Accessing PHM care and (5) Emotional sharing and coping strategies. Themes comprised sub-categories. Direct quotes cited from interviews or focus groups from the relevant included studies are used to illustrate the themes and their sub-categories.

#### Theme 1: Perceived causes of PMH

This theme comprises the perceived causes of PMH conditions such as depression and anxiety. The sub-categories include a range of different psychosocial, cultural and socioeconomic issues.

##### Lack of practical support

Four studies reported findings suggesting lack of practical support from in-laws and husbands in helping out with household chores to be a cause of PMH conditions [3, 9, 20, 21]. This view was found among postpartum women [3, 9], nurse practitioners [21] and family members of postpartum women [20]. This is illustrated by this quote cited from an interview with a first-time mother in a study conducted in India:

“I have to do everything by myself. I need someone to help me. I haven’t gone to stay with mother because then my husband will have trouble managing things; he will have to cook by himself. So I’ll go later and stay a month with her.” [3]

##### Poor relationship with husband/in-laws

Four studies found that a poor relationship with husband or in-laws was perceived to be a cause of PMH conditions [3, 19, 21, 22]. A quote cited from an interview with a postpartum mother in a study conducted in India highlights the importance women attach to the relationship with their husband and the care that he provides:

“Once you are married you are under the care of your husband. If your husband is not good then there is tension and you wonder why you ever got married.” [3]

##### Intimate partner violence (IPV)

Five studies reported IPV to be perceived as a cause of PMH conditions by pregnant [19] and postpartum women [3, 9, 19, 22] and family members of postpartum mothers [20]. IPV was found mainly to take the form of spousal emotional and physical violence both during and after pregnancy as highlighted in this quote from a postpartum mother in India.

“He used to hit me when I was pregnant. We had an argument and I said I was going to leave him. He said we should both die and save our lives. I said I would give back all the money he had spent on me. Then, he hit me badly; I fell on my stomach.” [3]

One study conducted in Bangladesh highlighted that along with spousal violence, women also feared violence from in-laws creating an abusive family environment for postpartum mothers [22].

“I am scared of them (the parents in-law), they look at me threateningly, and they want to pick issues. When they come home, I stay in my room and don’t get out.” [22]

##### Gender of the baby

Gender of the baby is a major concern for perinatal women in South Asian countries and having a female child has been highlighted as a perceived cause of PMH conditions in five studies [3, 19–22]. The stress caused by familial abuse when having a daughter is highlighted in this quote cited from a focus group discussion with postnatal women in a study conducted in India:

“This time also I have given birth to a girl child [and] because of this the family atmosphere is not good. My family members are abusing me and because of this I am going through a lot of stress.” [19]

Health care providers also stated that pressure from the husband and in-laws for a male child were causes of perinatal depression. This perception was more prominent in nursing providers than medical practitioners [21] as they tend to have more discussion time with perinatal women during antenatal and postnatal care visits.

##### Economic difficulties

Poverty was reported to be an important cause of PMH conditions by perinatal women and their family members [3, 9, 20, 22]. In rural areas, husbands often work as farmers on others’ lands which leads to unsteady income due to periods of unemployment [3]. For perinatal women, this results in worries around meeting medical costs during and after her pregnancy and being able to feed her baby properly. It also indicates their financial dependency on husbands.

“The main tension is because of the unemployment of my husband. Since it’s summer now, he can go anywhere (and do odd jobs), but once it starts raining, where can he go…my elder son has jaundice. If one of them is better, then the other one falls ill. I’m worried because we have to buy the medicine prescribed by the doctor.” [3]

##### Spiritual

Three studies reported perceptions of spiritual causes of PMH conditions. These views were found in perinatal women [17, 18] and family members [20]. Many thought that mental illness during pregnancy was caused by being possessed by God or some devil, committing sin, black magic or witchcraft.

#### Theme 2: Perceived symptoms of PMH

This theme comprises findings relating to the views of perinatal women, family members and health care providers on the symptoms and effects of PMH conditions.

##### Physical symptoms

Four studies highlighted that many perinatal women attached importance to physical, somatic signs such as weakness, backache, headache and “brain stops” when describing the symptoms and effects of PMH conditions [3, 9, 17, 22]. This is illustrated by this quote cited from an interview with a postpartum mother when asked about symptoms of depression in a study conducted in India:

“The headaches, dizziness, cold, tiredness all are the effects of this tension.” [3]

It was also suggested by Goyal et al. [17] that women tend to focus more on these physical symptoms when trying to describe poor PMH to others such as healthcare providers or their families, as women perceive these to be more acceptable and treatable symptoms rather than symptoms such as worry, fear, loneliness and stress. Physical symptoms prevent women from completing household chores or looking after her baby which was also highlighted to be a concern within perinatal women [22].

##### Emotional symptoms

However, some studies found that perinatal women and health care providers do consider emotional symptoms of PMH to be important [3, 17, 19, 21, 22]. Some women described symptoms of PMH as feeling sad, low mood, being depressed, crying, having tension or being mentally weak. These symptoms were acknowledged by women, but they would not always share these [17].

“Things are running in my head. I can’t stop worrying about everything.” [22].

Some pregnant women did not consider certain physical symptoms such as change in appetite, sleep problems and feeling tired as symptoms of depression [21]. Such symptoms are considered to be a normal part of pregnancy or of the postpartum period [19].

#### Theme 3: Perceptions of motherhood

Perceptions that individuals carry towards pregnancy and motherhood impacts the way communities view mental health issues within this population group as they have preconceived notions of what pregnancy should entail and how mothers should behave. In this theme, these perceptions are highlighted and how this impacts their attitudes towards PMH is explained.

##### Motherhood is sacred

Motherhood is sacred and an integral part of being a woman in many South Asian communities as highlighted by three studies [9, 20, 21]. When a woman becomes a mother, her status is elevated in her family and society and it is acknowledged as an accomplishment as stated by a postpartum mother in a study conducted in Bangladesh:

“Now everything is changed, people say that now you are a mother, they give me more importance, and they are so happy with me.” [9]

However, this places pressures on women to live up to this status. These societal expectations placed on mothers can result in criticism of women who may appear to struggle during pregnancy or postpartum, particularly to the point where she needs to access care [9]. In addition, these perceptions can be subject to certain conditions such as having female children which is often perceived as a status reducing characteristic for mothers [22].

##### Baby’s health is most important

Prominence is attached to a baby’s health as an important aspect of maternal wellbeing as highlighted in five studies [3, 9, 19, 20, 22]. Women during and after pregnancy assess their mothering abilities, health and success on the basis of their baby’s health.

“The most important thing is this that baby is healthy.” [19].

“I am more worried now about the baby…if my baby stays healthy, I will be in peace.” [22]

#### Theme 4: Accessing PMH care

This theme highlights some of the key barriers and enablers of access to PMH care as perceived by perinatal women, their family members and healthcare providers.

##### Mental health awareness

A key enabler in improving access to PMH care is professional awareness of mental health and the understanding that perinatal women are at risk of mental health conditions [3, 9, 18, 19, 21]. Many healthcare providers had poor awareness and understanding of mental health and PMH and perceived that such conditions were not a serious health concern [3, 9, 21]. This is highlighted in this quote cited from an interview with a gynaecological intern in a study conducted in Bangladesh:

“There is no mental health in maternal health – we don’t take mental health seriously, it’s just not there.” [9]

Awareness among perinatal women varies with many finding it difficult to understand the links between pregnancy or motherhood and depression.

“Why would she be sad? I don’t understand.” “I don’t know, this does not happen.” [9].

One study highlighted that the husbands of postpartum women have very limited awareness of PMH [3]. Husbands are less aware of the emotional needs of women during and after pregnancy and they often feel their only duty is to provide financial support [3]. Many husbands of women with postpartum depression were also ignorant that their wives were unwell in any way [3].

##### Stigma and shame

Stigma and the related shame associated with PMH in Bangladesh, India and Pakistan is a key barrier for perinatal women accessing PMH care as highlighted by seven studies [3, 9, 17–21]. There is the attitude that emotional health problems should stay within the family and women would not tell their friends if they do attend mental health services because of the taboo attached to them [9].

Attitudes of family members of perinatal women also reflect these stigmatisations regarding women with postpartum depression characterising them as not being good mothers [20]. Nevertheless, many are ready to help reduce depression through family interventions. Nursing providers stated that postpartum depression is a sign of weakness and “women choose to get postpartum depression.” [21].

Stigma results in women being ashamed or fearful to access care due to repercussions from family or judgment from healthcare providers and communities [19]. These concerns are highlighted in this quote cited from a focus group discussion with pregnant women in a study conducted in Pakistan:

‘Some women will not talk about their problems as they are afraid. They think that their husband will know about it if they talk to doctor and will be angry with them.” [19]

##### Friendly and confidential counselling

McCauley et al. [19] found that perinatal women would find counselling acceptable if healthcare providers providing the counselling were friendly, kept the information confidential and removed the stigma attached to PMH. Counselling is a more appropriate form of treatment compared to drugs and medications for healthcare providers to offer and perinatal women to receive [20, 21].

Counselling and social support have been acknowledged as helpful by many women but require much more cultural attention to tackle the shame and stigma associated with it [17]. Western protocols and counselling methods may not work as well in these South Asian settings as they need to consider religious, cultural and societal beliefs which shape a lot of the beliefs around PMH [3, 9].

##### Limited resources

A key barrier in accessing adequate PMH care is the limited resources in these South Asian countries as reported by five studies [3, 9, 17–19]. One aspect of limited resources is the lack of time that healthcare providers have which prevents them from focusing on PMH [9, 19].

“A doctor has many patients…obviously, they don’t have time for these details!” [19].

Another element is poverty and lack of money in the health system to treat PMH conditions and within families to access such treatments [3, 17, 18]. Resources are very constrained within the healthcare system, with respect to mental health, and healthcare providers have to make choices about the care they deliver and physical health takes precedence [9].

“How can we focus on maternal depression when unattended birth and postpartum haemorrhage is such a problem, and we are a poor country?” [9]

There is also limited education for healthcare providers on PMH with counselling not widely taught and no protocols on how to educate or identify women with postpartum depression [9]. This lack of education impacts the care available to perinatal women.

#### Theme 5: Emotional sharing and coping strategies

This theme highlights the ways in which perinatal women share their emotions and the “coping” strategies (term used by William et al. [9]) they adopt to manage their PMH conditions.

##### Sharing is pointless

Many women reported that sharing their mental health issues is pointless or difficult because of resulting quarrels and judgment from others. [3, 9, 19] Family members tend to be disinterested in such matters and women feel that healthcare providers will not be able to do much as these are family related problems [3]. Hence, emotional sharing tends to be very limited in these South Asian countries.

“It’s not necessary to share, I feel better to just keep things to myself. I am afraid of sharing.”[9]

##### Personal coping strategies

With limited emotional sharing with others, perinatal women tend to resort to personal coping strategies [3, 9, 17]. A common form is through distraction from the issues that worry women by getting out of the house or playing with their baby [9]. This is highlighted by this quote cited from an interview with a Bangladeshi postpartum mother when asked how she copes with stress:

“Sometimes I go outside the gate and stand there, and then I come back.” [9].

##### Religious coping strategies

Aside from coping through personal strategies, four studies, Rodrigues et al. [3], Williams et al. [9], Goyal et al. [17] and Manjrekar et al. [18] found that perinatal women would also turn to religious coping methods through prayers, faith-healing and crying for God’s help. Religion is often a source of comfort and strength for many women in these South Asian countries.

## Discussion

This review synthesised data from primary studies investigating perceptions and attitudes around PMH in Bangladesh, India and Pakistan, a topic largely overlooked in the literature to date. This review identified five themes: perceived causes of PMH, perceived symptoms of PMH, perceptions of motherhood, accessing PMH care and emotional sharing and coping strategies.

The perceptions and attitudes identified in this review are largely related to sociocultural factors, reflecting cultural collectivism and the local social construction of gender roles and power relations. This is evident from the perceived causes of PMH conditions as identified from the studies included in this review. These findings relate to evidence from cross-sectional studies which found significant associations between IPV [23–26], poor relationship with husband/in-laws [8, 26], low practical support [25, 26], female gender of the baby [5, 24, 27], and antenatal depression among women in South Asia. Social construction of gender roles within South Asian communities often places women at a disadvantage. Women are expected to carry out all the household chores, even during pregnancy, with little practical support and an inability to do this can result in repercussions from the family including being subjected to violence [24]. Even before a woman is born, society deems her to be of lower status and value than men as reflected by the preference for male children [28]. These gender inequalities are rooted within many South Asian communities and can strongly influence individuals’ perceptions and attitudes.

Affective symptoms of PMH are acknowledged by perinatal women, however, discussing physical, more somatic symptoms is much more acceptable due to the perception that women with mental health conditions are ‘weak’ or ‘mad’. This is common in Asian culture as highlighted in the review by Halbreich & Karkun [4] who investigated cross-cultural postpartum depression prevalence and symptoms. Since motherhood is sacred, is it not socially acceptable to be seen as weak. Thus, women are forced to hide their emotional symptoms from family members and healthcare providers with the fear that their child may be removed from their care. Even healthcare providers’ attitudes are dominated by collectivist cultural controls where they infrequently ask about emotional or mental health symptoms as they perceive it to be inappropriate. These findings are consistent with the systematic review by Watson et al. [11] on South Asian and Black African women’s experiences of PMH in the UK.

Barriers and enablers to accessing PMH care operate at a number of levels ranging across sociocultural to structural factors. Perinatal women who acknowledged mental health conditions were more open to discussing these with healthcare providers. Family members and healthcare providers who had adequate awareness around the importance of PMH also enabled perinatal women to access care. This is consistent with other studies which found that lack of awareness among women, their families and healthcare providers led to poor recognition of symptoms and delayed management [11, 13]. Therefore, increased training and education is required to increase awareness around PMH.

In addition, lack of awareness often stems from the high levels of stigma associated with mental health which is a significant barrier for accessing PMH care. This again highlights the theory of collectivist cultures [29] where societal stigma found within these South Asian countries dominate the attitudes people have towards PMH resulting in women who access PMH care being criticised. These findings are highlighted in previous literature where stigma is a key barrier to accessing PMH care [11, 30]. Women do not want to be labelled with mental health conditions as society will deem them to be weak and unsuitable mothers. This relates to the emotional sharing and coping strategies adopted by perinatal women in this review. Sharing is perceived as pointless as they won’t be taken seriously by a society which belittles PMH, therefore, women resort to personal and religious coping strategies [3, 9, 17]. Distraction and praying are two methods; however, these are not sustainable. This calls for culturally appropriate coping interventions for long-term management.

Structural factors such as limited resources also create barriers for perinatal women receiving PMH care and healthcare providers delivering the care. Lack of time and limited health workforce means healthcare providers are forced to focus only on physical health. Postpartum haemorrhage is a leading cause of maternal mortality in South Asian countries [31]. Therefore, immediate attention must be given to such physical health concerns. Poverty plays a role in the limited resources in countries such as Bangladesh who only spend 0.5% of the health budget on mental health [32]. There is limited money to spend on mental health services and facilities which are accessible for perinatal women from all regions. These findings are consistent with other LMICs [30].

These findings have implications for informing cross-sectoral prevention and management interventions for PMH in these South Asian countries. The education sector will be a key actor in the development of these interventions addressing many of the perceptions and attitudes identified in this review. Improved education for healthcare providers at all levels is vital for increasing awareness of PMH and better knowledge of the symptoms and management options [33]. Healthcare providers working directly with perinatal women should be given adequate training around friendly and confidential counselling. Psychosocial education aimed to tackle IPV, gender inequalities and stigma within communities could be provided through Government and non-governmental social programmes. Family planning services are also key to address the issue of unplanned pregnancies. This can be achieved through Maternal, Neonatal and Child Health platforms who can provide these services at a community and household level through community health workers who are more aware of the rural settings and culture. The Social welfare sector should be integrated to tackle poverty, housing and unemployment, for example, by encouraging microcredits for women to start their own small businesses and promoting health knowledge [34].

To our knowledge this is the first systematic review of literature focusing on perceptions and attitudes around PMH in Bangladesh, India and Pakistan. A wide range of databases were searched using a comprehensive search strategy and supplementary searches were carried out. However, we acknowledge some possible limitations. Due to resource constraints, articles were restricted to those in the English language which may produce language bias. Nevertheless, journals from Bangladesh, India and Pakistan tend to increasingly publish in the English language or contain translations making them accessible for a wider audience. Second, although most studies were deemed to be of ‘adequate’ or ‘medium’ quality, there were gaps in reporting, particularly with regards to possible researcher bias during data collection and analysis and what implications this may have on the findings and conclusions in the included studies. Finally, only eight studies were identified for inclusion in this review, with only one from Pakistan which may question the relevance of the themes in a Pakistani setting. Nevertheless, qualitative reviews adopt more interpretative approaches using depth and richness of data rather than breadth which privilege smaller number of studies [35]. Given this, Booth et al. [35] suggests a preferred number of between 6 and 14 studies for a qualitative review.

## Conclusion

There is a complex range of perceptions and attitudes around PMH which influence women’s experiences and access to PMH care within Bangladesh, India and Pakistan. Sociocultural expectations underpin many of the perceptions and attitudes identified in this review including the importance of familial and societal causes of PMH, emphasis on physical symptoms, sacredness of motherhood, lack of awareness, stigma, shame, limited resources allocated for mental health and lack of emotional sharing. These interlinking perceptions reflect the cultural collectivist nature of South Asian cultures preventing proper identification and adequate care for perinatal women with PMH conditions. These findings can be used to inform policy and practice through targeted interventions to tackle stigmatising attitudes and increasing education and training for healthcare providers. Further research should explore perceptions and attitudes of perinatal women along with key family members, such as husband and mother-in-law, and community-based healthcare providers in Bangladesh, where there is limited qualitative research, particularly during the antenatal period.

## Supplementary Information

**Additional file 1.** The ENTREQ checklist.

**Additional file 2.** Search terms for OVID database.

**Additional file 3.** Summary of CASP tool used for quality appraisal of qualitative studies.

**Additional file 4.** Summary of NOS tool used for quality appraisal of cross-sectional studies.

## Data Availability

All data from the included studies analysed in this review are publicly available and included in this published article.

## Abbreviations

CASP: Critical Appraisal Skills Programme
IPV: Intimate Partner Violence
HICs: High Income Countries
LMICs: Lower- and Middle-Income Countries
NOS: Newcastle-Ottawa Scale
PMH: Perinatal Mental Health
