# Supplementary material for: Perceptions and attitudes around perinatal mental health in Bangladesh, India and Pakistan: a systematic review of qualitative data
Source: BMC Pregnancy Childbirth. 2022 Apr 6;22:293. doi: 10.1186/s12884-022-04642-x (PMC8988352; doi:10.1186/s12884-022-04642-x)
Supplement: Supplementary file 2 — Additional file 2. Search terms for OVID database. [file 12884_2022_4642_MOESM2_ESM.docx]

**Search terms* for OVID database**

1. perspective*.mp.
2. views.mp.
3. opinions.mp.
4. attitude*.mp.
5. perception*.mp.
6. factors*.mp.
7. "risk factors".mp.
8. determinants.mp.
9. dimensions.mp.
10. correlates.mp.
11. 1 or 2 or 3 or 4 or 5 or 6 or 7 or 8 or 9 or 10
12. Maternal Health/ or maternal.mp.
13. perinatal.mp.
14. antenatal.mp. or Pregnancy/
15. pregnan*.mp.
16. postpartum.mp.
17. women.mp.
18. 12 or 13 or 14 or 15 or 16 or 17
19. depress*.mp.
20. Depression, Postpartum/
21. Anxiety/ or anxiety.mp.
22. anxi*.mp.
23. stress.mp.
24. mood.mp.
25. 19 or 20 or 21 or 22 or 23 or 24
26. Bangladesh/ or bangladesh*.mp.
27. Pakistan/ or pakistan*.mp.
28. India/ or india*.mp.
29. "south asia*".mp.
30. 26 or 27 or 28 or 29
31. 11 and 18 and 25 and 30

*This search strategy combined two reviews, the other review investigated the social determinants of antenatal depression in Bangladesh, India and Pakistan. See PROSPERO protocol CRD42020167903.
