# Supplementary material for: Perceptions and attitudes around perinatal mental health in Bangladesh, India and Pakistan: a systematic review of qualitative data
Source: BMC Pregnancy Childbirth. 2022 Apr 6;22:293. doi: 10.1186/s12884-022-04642-x (PMC8988352; doi:10.1186/s12884-022-04642-x)
Supplement: Supplementary file 3 — Additional file 3. Summary of CASP tool used for quality appraisal of qualitative studies. [file 12884_2022_4642_MOESM3_ESM.docx]

| Author/year | Clear Aims | Appropriate methodology | Appropriate research design | Appropriate recruitment strategy | Appropriate data collection methods | Researcher-participant relationship considered | Ethical issues considered | Rigorous data analysis | Clear findings | Value of research | Overall assessment |
| --- | --- | --- | --- | --- | --- | --- | --- | --- | --- | --- | --- |
| Edhborg et al, 2015 | Y | Y | Y | Y | Y | ? | Y | ? | Y | Y | Adequate |
| McCauley et al, 2020 | Y | Y | Y | Y | Y | ? | ? | Y | Y | Y | Adequate |
| Rodrigues et al, 2003 | Y | Y | Y | Y | Y | ? | ? | ? | Y | Y | Adequate |
| Williams et al, 2018 | Y | Y | Y | ? | Y | ? | Y | Y | N | Y | Adequate |

**Summary of CASP tool used for quality appraisal of qualitative studies**

Y= Yes

? = Insufficient information to make an assessment

N = No

Scoring

A scoring of 1 mark for each question that was answered with “Y” was allocated to provide an indicator of quality and enable comparison between reviewers. Cut-off scores of $\geq$9 indicated ‘strong’ quality, 6-8 indicated ‘adequate’ quality and $\leq$5 indicated ‘weak’ quality for the CASP tool.
