# Supplementary material for: Perceptions and attitudes around perinatal mental health in Bangladesh, India and Pakistan: a systematic review of qualitative data
Source: BMC Pregnancy Childbirth. 2022 Apr 6;22:293. doi: 10.1186/s12884-022-04642-x (PMC8988352; doi:10.1186/s12884-022-04642-x)
Supplement: Supplementary file 4 — Additional file 4. Summary of NOS tool used for quality appraisal of cross-sectional studies. [file 12884_2022_4642_MOESM4_ESM.docx]

**Summary of NOS tool used for quality appraisal of cross-sectional studies**

| **Paper** | **Newcastle Ottawa Scale question number and score allocated^a^** | | | | | | | |  |
| --- | --- | --- | --- | --- | --- | --- | --- | --- | --- |
|  | **1** | **2** | **3** | **4** | **5** | **6** | **7** | **Total stars^b^** | **Category^c^** |
| Goyal et al, 2020 | b* | a* | a* | b* | c | a** | b | 6 | Medium |
| Manjrekar et al, 2018 | b* | b | c | b* | c | c* | b | 3 | Low |
| Poreddi et al, 2020 | a* | b | a* | b* | c | a** | a* | 6 | Medium |
| Ransing et al, 2020 | b* | b | c | a** | c | c* | a* | 5 | Medium |

^a^Newcastle-Ottawa question numbers 1-7, answers a-d, and associated number of stars (*) are detailed below

^b^Minimum number of possible stars to be awarded = 0, maximum number of possible stars to be awarded = 10.

^c^Categories were allocated as: Low = 0-3 stars, Medium = 4-7 stars, High = 8-10 stars.

**Selection (maximum 5 stars)**

1. Representativeness of the sample
   1. Truly representative of the average in the target population/sampling frame (all subjects or random sampling) *
   2. Somewhat representative of the average in the target population/sampling frame (non-random sampling) *
   3. Selected group of pregnant women
   4. No description of sampling strategy
2. Sample size
   1. Justified and satisfactory *
   2. Not justified
3. Non-respondents
   1. Proportion of target sample recruited attains pre-specified target or basic summary of non-respondent characteristics in sampling frame recorded. *
   2. Unsatisfactory recruitment rate, no summary data on non-respondents.
   3. No description of the response rate or the characteristics of the respondents and the non-respondents
4. Ascertainment of the exposure (risk-factors)
   1. Validated questionnaires used/record linkage **
   2. Non-validated questionnaires, but the questions are available or described. *
   3. No description of the measurement tool

**Comparability^a^ (maximum 2 stars)**

1. The subjects in different outcome groups are comparable, based on the study design or analysis. Confounding factors are controlled.
   1. The study controls for the important factors (as identified by univariate analysis in the study or justified). **
   2. The study controls for factors but no justification as to why they were chosen. *
   3. No factors controlled for.

**Outcome (Depression/anxiety) (maximum 3 stars)**

1. Assessment of the outcome^b^
   1. Validated measurement tool with depression/anxiety categories justified and appropriate **
   2. Record linkage **
   3. Validated measurement tool but depression/anxiety categories not justified. *
   4. No description of ascertainment method.
2. Statistical analysis
   1. The statistical test used to analyze the data is clearly described and appropriate, and the measurement of the association is presented, including confidence intervals and the probability level (p value). *
   2. The statistical test is not appropriate, not described, or incomplete

This has scale has been adapted from the Newcastle-Ottawa Quality Assessment scale for cohort studies to provide quality assessment for cross-sectional studies.^1^

^a^We have not selected one factor that is the most important for comparability, because the variables are not the same in each study. Thus, the study should justify why they have controlled for certain factors like if they were significant in univariate analysis.

^b^Original question 6 options: a) independent blind assessment b) record linkage c) self-report d) no description, are not applicable to this study as depression/anxiety were the outcomes of interest which are usually measured using a depression/anxiety scale which in nature contain self-reported questions. Therefore, the options were adapted to suit this systematic review.

**Reference**

1. Herzog R, Alvarez-Pasquin MJ, Diaz C, Del Barrio JL, Estrada JM, Gil A. Are healthcare workers’ intentions to vaccinate related to their knowledge, beliefs and attitudes? A systematic review. *BMC Public Health*. 2013. 154. <https://doi.org/10.1186/1471-2458-13-154>
